# Allergic Reactions After the Administration of COVID-19 Vaccines

**DOI:** 10.3389/fpubh.2022.878081

**Published:** 2022-05-17

**Authors:** Sainan Bian, Lisha Li, Zixi Wang, Le Cui, Yingyang Xu, Kai Guan, Bin Zhao

**Affiliations:** ^1^Department of Allergy, Peking Union Medical College Hospital, Peking Union Medical College, Chinese Academy of Medical Sciences, Beijing, China; ^2^Peking Union Medical College, Beijing Key Laboratory of Precision Medicine for Diagnosis and Treatment of Allergic Disease, Beijing, China; ^3^National Clinical Research Center for Dermatologic and Immunologic Diseases (NCRC-DID), Beijing, China; ^4^Pharmacy Department, Peking Union Medical College Hospital, Peking Union Medical College, Chinese Academy of Medical Sciences, Beijing, China

**Keywords:** allergic reaction, anaphylaxis, COVID-19 vaccine, Vaccine Adverse Event Reporting System (VAERS), vaccination

## Abstract

**Background:**

Data on allergic reactions after the administration of coronavirus disease (COVID-19) vaccines are limited. Our aim is to analyze reports of allergic reactions after COVID-19 vaccine administration.

**Methods:**

The Vaccine Adverse Event Reporting System database was searched for reported allergic reactions after the administration of any of the COVID-19 vaccines from December 2020 to June 2021. After data mapping, the demographic and clinical characteristics of the reported cases were analyzed. Potential factors associated with anaphylaxis were evaluated using multivariable logistic regression models.

**Results:**

In total, 14,611 cases were reported. Most cases of allergic reactions comprised women (84.6%) and occurred after the first dose of the vaccine (63.6%). Patients who experienced anaphylaxis were younger (mean age 45.11 ± 5.6 vs. 47.01 ± 6.3 years, *P* < 0.001) and had a higher prevalence of a history of allergies, allergic rhinitis, asthma, and anaphylaxis than those who did not (*P* < 0.05). A history of allergies (odds ratio (OR) 1.632, 95% confidence interval (CI) 1.467–1.816, *P* < 0.001), asthma (OR 1.908, 95%CI 1.677–2.172, *P* < 0.001), and anaphylaxis (OR 7.164, 95%CI 3.504–14.646, *P* < 0.001) were potential risk factors for anaphylaxis. Among the 8,232 patients with reported outcomes, 16 died.

**Conclusions:**

Female predominance in allergic reaction cases after the receipt of COVID-19 vaccines was observed. Previous histories of allergies, asthma, or anaphylaxis were risk factors for anaphylaxis post-vaccination. People with these risk factors should be monitored more strictly after COVID-19 vaccination.

## Introduction

The severe acute respiratory syndrome coronavirus 2 (SARS-CoV-2) is still pandemic globally and the number of patients with coronavirus disease (COVID-19) is increasing. As of October 2021, the number of accumulated confirmed cases is approximately 235 million, with 4.8 million deaths. To date, the disease remains endemic, and the number of confirmed cases continues to increase. The development of COVID-19 vaccines has brought new hope to combat the virus. The newly updated (October 1 2021) COVID-19 vaccine tracker and landscape of the World Health Organization showed that the number of COVID-19 vaccine candidates in both clinical and pre-clinical development was 317 (1). Several COVID-19 vaccines have been approved by the United States Food and Drug Administration (FDA) for emergency use since December 2020.

The Pfizer-BioNTech (BNT162b2) vaccines against COVID-19 are mRNA-based vaccines with lipid nanoparticle-encapsulated, and encode the prefusion-stabilized full-length spike protein of SARS-CoV-2. The Moderna (mRNA-1273) vaccines are also mRNA-based vaccines. The Janssen Ad26.COV2.S vaccine comprises a recombinant, replication-incompetent adenovirus serotype 26 (Ad26) vector, and encodes a stabilized full-length spike protein of SARS-CoV-2. Randomized controlled trials of these vaccines showed a low rate (0.4–0.6%) of severe side effects (2–4).

Adverse events, including allergic reactions, have been reported after administration of the first dose of a COVID-19 vaccine (5–7). However, studies on allergic reactions after large-scale administration of COVID-19 vaccines are limited. In this study, we aimed to summarize reports of allergic reactions after COVID-19 vaccine administration according to the Vaccine Adverse Event Reporting System (VAERS) database.

## Methods

### Data Source and Data Mining

Data for this study were based on the VAERS database. The VAERS database is operated by the FDA and Centers for Disease Control and Prevention (CDC). It was set up in 1990, used as a vaccine safety surveillance system of the United States (internet address: https://vaers.hhs.gov). Adverse events after administration of vaccines are collected and reported to the VAERS as an early warning. These adverse events are reported by vaccine recipients, healthcare workers, and vaccine manufacturers. If the adverse events of vaccines are regarded as contraindications to further doses, vaccine manufacturers and healthcare workers are required to report the adverse events by law. With this way, the opportunity of ignoring vital and relating adverse events is reduced. All adverse events possibly related to vaccines are collected in VAERS database. However, these adverse events are not determined of clinical significance or whether are caused by the vaccine. Even though, VAERS is still of vital significance as a hypothesis-generating system with the original target of detecting adverse events possibly associated with vaccines (8, 9).

Several files are included in the VAERS data: VAERSDATA.CSV (contains reports and patient information), VAERSVAX.CSV (contains vaccine information), and VAERSSYMPTOMS.CSV (contains adverse event information). The file of VAERSDATA.CSV includes demographic information; past medical history; and a history of allergies (allergies to medications, food, or other products). The file of VAERSVAX.CSV includes manufacturers and providers of the vaccine. The file of VAERSSYMPTOMS.CSV includes the date when vaccines received, date when symptoms occurred, symptom description, and outcome of the reported cases. Adverse events descriptions were coded using an internationally standardized, clinically validated terminology, the preferred terms (PTs) of the Medical Dictionary for Regulatory Activities (MedDRA) dictionary. Reports on COVID-19 vaccines in the VAERS database were included until 11 June 2021. The data were retrieved from the 2021 Zip File of VAERS Data Sets and were downloaded on 18 June 2021.

The study was approval by the Institutional Review Board of Peking Union Medical College Hospital (S-K1810).

### Allergic Reactions Mapping

MedDRA Version 22.1 was used for allergic reaction mapping. The following terms were considered allergic reactions when COVID-19 vaccines were administered and these cases were included: “urticaria,” “angioedema,” “anaphylaxis,” “anaphylactic shock,” and “anaphylactic reaction.” Reports were identified using the above PTs. Reports including the following PTs that possibly were consistent with the Brighton Criteria case definition for anaphylaxis were also identified: “acute urticaria,” “acute angioedema,” “pharyngeal swelling,” “throat tightness,” “dysphonia,” “respiratory distress,” “hypoxia,” “cough,” “wheezing,” “dyspnea,” “vomiting,” “diarrhea,” “hypotension,” “loss of consciousness,” “mental status changes,” “syncope,” “incontinence,” “altered state of consciousness.”

Anaphylaxis was defined based on the World Allergy Organization (WAO) anaphylaxis guidance (10). The available description of symptoms was reviewed to identify if the symptoms were accorded with the Brighton Collaboration case definition for anaphylaxis (11) or under the diagnosis of anaphylaxis by a physician.

Grading of systemic acute allergic reactions was based on WAO guidance (10).

Ratio of allergic reactions of different types of vaccines was compared.

This study was followed by the GATHER guidelines (12).

### Data Statistical Analysis

Descriptive analysis was used to describe the clinical and demographic characteristics of patients with acute allergic reactions after COVID-19 vaccine immunization in the VAERS database. Data of normal distribution are expressed as mean±standard deviation (SD), and data of non-normal distribution are expressed as median and interquartile range (IQR). Continuous and categorical variables were compared using the independent samples *t*-test and Pearson χ2 test or Fisher's exact probability test, respectively. Non-parametric tests were used for non-normally distributed data. Potential factors associated with anaphylaxis were evaluated using multivariable logistic regression models. *P* < 0.05, with 95% confidence intervals (CI), indicated statistical significance. Statistical analysis was conducted using SPSS 22.0 (SPSS Inc, Chicago, IL, USA).

## Results

### Characteristics of Cases With Allergic Reactions

A total of 14,611 cases were reported, with the highest in April 2021(3,526, 24.1%). Most cases of allergic reactions were of women (84.6%), comprised the 18–44-year age group (47.4%), had allergic reactions after the first dose of COVID-19 vaccination (63.6%), and occurred on the day of vaccine administration (41.9%) (Table 1).

**Table 1:** Demographic characteristics and vaccine information of cases with hypersensitivity reactions after COVID-19 vaccine reported to VAERS database.

**Characteristics**	**Reports, n (%)**
Reporting date	
December 2020	568 (3.9)
January 2021	3,454 (23.6)
February 2021	2,427 (16.6)
March 2021	3,108 (21.3)
April 2021	3,526 (24.1)
May 2021	1,020 (7.0)
June 2021	508 (3.5)
Gender of reported cases	
Male	2,230/14,440 (15.4)
Female	12,210/14,440 (84.6)
Unknown or missing	171/14,611 (1.2)
Age groups (years)	
<18	210/14,143 (1.5)
18–44	6,706/14,143 (47.4)
45–64	4,847/14,143 (34.3)
≥65	2,380/14,143 (16.8)
Unknown or missing	468/14,611 (3.2)
Vaccine producer	
Janssen	987 (6.8)
Moderna	7,525 (51.5)
Pfizer-Biontech	6,070 (41.5)
Unknown	29 (0.2)
Dosage	
First dose	9,296 (63.6)
Second dose	3,412 (23.4)
Unknown or missing	1,903 (13.0)
Onset interval (days)	
0 day	6,117 (41.9)
1 day	2,192 (15.0)
2 days	1,061 (7.3)
3–7 days	2,567 (17.6)
>7 days	2,048 (14.0)
Unknown or missing	626 (4.3)

*Onset interval: the interval between the day when the vaccine administrated and the day when symptom occurred*.

### Comparison of Cases With Anaphylaxis and Non-anaphylaxis Allergic Reactions

Among the 14,611 reported cases, 3,225 (22.1%) were of anaphylaxis. Most were skin allergic reactions (77.9%), with no other organs involved. Patients with anaphylaxis were younger (45.11 ± 5.6 vs. 47.01 ± 6.3 years, *P* < 0.001); more likely to be women (86.5 vs. 84.0%, *P* = 0.001); and more likely to have a history of allergies, allergic rhinitis, asthma, or anaphylaxis (*P* <0.05) than those without anaphylaxis (Table 2).

**Table 2:** Comparison of reported cases with anaphylaxis and nonanaphylaxis allergic reactions.

**Characteristics**	**Anaphylaxis** **(*n* = 3225, 22.1%)**	**Nonanaphylaxis allergic reactions** **(*n* = 11386, 77.9%)**	***P*** **-value**
Age-year (mean±SD)	45.11 ± 5.6	47.01 ± 6.3	<0.001^*^
Female-n (%)	2,727/3,152 (86.5)	9,483/11,288 (84.0)	0.001^*^
Previous history			
Allergies^†^	1,770 (54.9)	5,856 (51.4)	0.001^*^
Allergic rhinitis	25 (0.8)	48 (0.4)	0.012^*^
Hay fever	4 (0.1)	12 (0.1)	1.000
Asthma	456 (14.1)	910 (8.0)	<0.001^*^
Anaphylaxis	31 (1.0)	13 (0.1)	<0.001^*^
Eczema	20 (0.6)	79 (0.7)	0.653
Chronic urticaria	4 (0.1)	9 (0.1)	0.673

*SD, standard deviation;*

* *p < 0.05;*

† *Allergies to medications, food, or other products*.

Among cases with anaphylaxis reported, 256 cases (7.9%), 1,323 cases (41.0%), and 1,636 cases (50.7%) received Janssen Ad26.COV2.S, mRNA-1273, and BNT162b2 vaccine, respectively. Among cases with non-anaphylaxis, 731cases (6.4%), 6,202 cases (54.5%), and 4,434 cases (38.9%) received Janssen Ad26.COV2.S, mRNA-1273, and BNT162b2vaccine, respectively. The differences were all significant (*p* < 0.001).

### Risk Factors for Anaphylaxis

**Figure 1:**
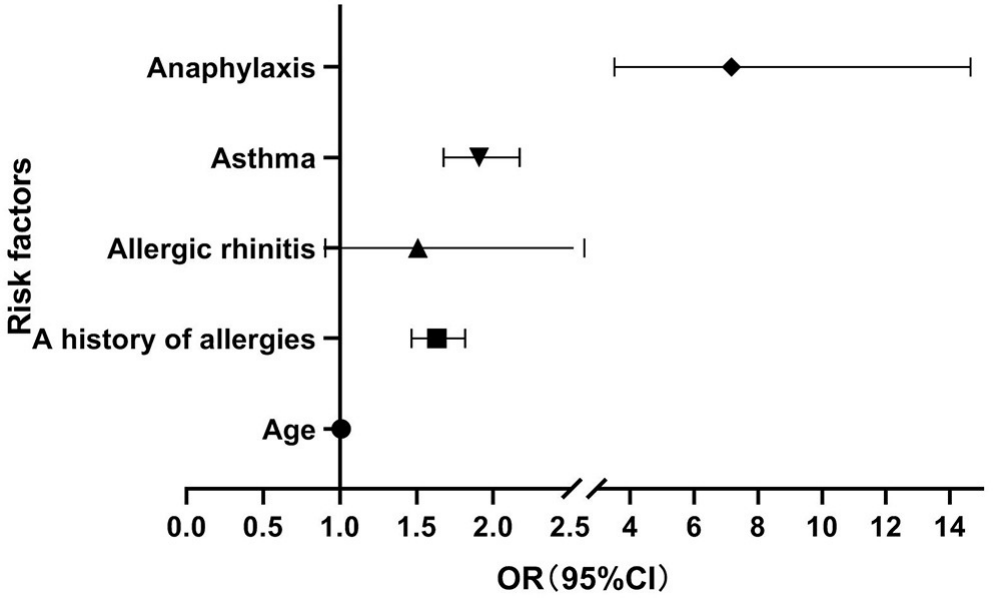
Risk factors for anaphylaxis.

Age and a history of allergies, allergic rhinitis, asthma, and anaphylaxis were included in the logistic regression analysis. A history of allergies (OR 1.632, 95%CI 1.467–1.816, *P* < 0.001), asthma (OR 1.908, 95%CI 1.677–2.172, *P* < 0.001), and anaphylaxis (OR 7.164, 95%CI 3.504–14.646, *P* <0.001) were potential risk factors for anaphylaxis after vaccination. Allergic rhinitis was not a risk factor (OR 1.508, 95%CI 0.904–2.515, *P* = 0.115). A history of anaphylaxis had the largest OR (Figure 1).

### Grading of Reported Systemic Allergic Reactions

Among the cases with allergic reactions, 10,141 (69.4%) had grade 1 allergic reactions with only generalized urticaria, 2,315 (15.8%) had grades 3–4 allergic reactions with respiratory and/or digestive tract involvement, and 230 (1.6%) had grade 5 allergic reactions with anaphylactic shock. There was no available description of symptoms for the 680 cases only reported as anaphylaxis (Table 3).

**Table 3:** Grading of systemic allergic reactions according to the WAO systemic allergic reaction grading system.

	**Reports (*n*)**
Grade 1–2	10,141
Grade 3–4	2,315
Pharyngeal swelling	392
Throat tightness	528
Dysphonia	126
Respiratory distress	36
Hypoxia	13
Cough	299
Wheezing	222
Dyspnoea	1,138
Vomiting	344
Diarrhea	273
Grade 5	230
Hypotension	66
Loss of consciousness	85
Mental status changes	4
Syncope	91
Incontinence	2
Altered state of consciousness	5
NA	680

*NA, Not available as no specific description of symptoms*.

### Outcome of Patients With Hypersensitivity Reactions

Among the 8,232 patients with reported outcomes, 16 died, 404 had life-threatening reactions, 3,185 visited an emergency room, 442 were hospitalized, and 52 were disabled. The median number of hospitalized days was 2.0 (IQR 1.0–3.0) days. At the time of reporting to VAERS, 39.8% of the patients had recovered and 39.0% had not recovered (Table 4). Among the 16 patients who died, 11 (68.8%) had grade 5 allergic reactions (Supplemental Table 1), and the other five were unavailable to undergo grading.

**Table 4:** Outcome of cases with hypersensitivity reactions.

	**Total N**	**Recovered-*N* (%)**	**Not recovered-*N* (%)**	**Unknown-*N* (%)**
Total	8,232	3,099 (37.6)	3,305 (40.1)	1,828 (22.2)
Clinic visit	4,133	1,351 (32.7)	2,067 (50.0)	715 (17.3)
ER visit	3,185	1,384 (43.5)	881 (27.7)	920 (28.9)
Death	16	0	16 (100.0)	0
Life-threatening	404	199 (49.3)	135 (33.4)	70 (17.3)
Hospitalized	442	158 (35.7)	169 (38.2)	115 (26.0)
Disabled	52	7 (13.5)	37 (71.2)	8 (15.4)

*ER, emergency room, N: number*.

## Discussion

We conducted a study on allergic reactions after COVID-19 vaccination as a complement to clinical trials. In this study, we summarized the characteristics of cases with allergic reactions, risk factors for anaphylaxis, and prognosis of patients with allergic reactions.

Immunologically mediated allergic reactions can cause various manifestations ranging from skin disorders to life-threatening systemic reactions. The allergic reactions caused by vaccines can be the following pathophysiologic mechanisms. They can be caused by activation of mast cells via interaction with immunoglobin E (IgE) antibodies. Activation of the complement system can lead to Non-IgE-mediated mast cell degranulation. Direct activation of the Mas-related G protein-coupled receptor X2 can also cause allergic reactions. Type IV hypersensitivity is cell-mediated and generally cause delayed reactions (13, 14).

In this study, 84.6% of the patients with allergic reactions were women. An early study reported of 21 patients who were diagnosed of anaphylaxis after administration of Pfizer-BioNTech COVID-19 vaccine. It also indicated a female predominance (7). Studies that summarized allergic reactions after the administration of other vaccines also reported a female predominance (13). It is also possible that a greater proportion of allergic reactions in females partly because a greater proportion of vaccines was administered to females than to males. However, whether the sex difference in the development of post-vaccination hypersensitivity reactions was due to the function of sex hormones or other elements remains unknown.

More than half of the reactions occurred after the first vaccination dose, highlighting the importance of monitoring people who receive the first dose of the COVID-19 vaccine. Another study about the adverse effects following immunization also showed that 79.68% of adverse effects occurred after the first dose (9). However, in a recent study of the safety of mRNA vaccines showed over half participants reported local and systemic reactogenicity more frequently after dose two than after dose one (15). Maybe longer observations are needed to confirm this.

Although differences were found among different vaccines of allergic reactions reported, this only represent the difference of reports to the VAERS. As the total number of people received different vaccines was unknown in this database, we couldn't know the ratio of allergic reactions among different vaccines.

Past histories of allergies; allergic rhinitis; asthma; and anaphylaxis were more common in patients with anaphylaxis than in those without anaphylaxis. Further logistic regression showed that a history of allergies; asthma; and anaphylaxis were risk factors for anaphylaxis post-COVID-19 vaccination. Other studies have also reported a high proportion of patients with a history of allergies in cases of anaphylaxis. According to a previous study of 21 cases of anaphylaxis after the administration of Pfizer-BioNTech mRNA vaccine, 17 had a past history of hypersensitivity reactions and seven had a past history of anaphylaxis (16). In a subsequent report of 10 cases of anaphylaxis after receiving the first dose of the Moderna mRNA vaccine until 10 January 2021, nine had a past history of hypersensitivity reactions and five had a past history of anaphylaxis (6). According to the American Academy of Allergy Asthma and Immunology (AAAAI) (6), approximately 30% of the general population has some kind of hypersensitivity or past histories of hypersensitivity reactions. Thus, we can see that cases with allergic reactions had a relatively higher ratio of a history of allergies than the general population. Individuals with a past history of allergies should be observed more strictly.

Of all patients, 16 (0.1%) died, of whom 68.8% had grade 5 allergic reactions with anaphylactic shock. In a previous report, 20 (95%) patients had hospital discharge or had recovery at the time of reporting adverse events to VAERS. There were no reports of deaths due to anaphylaxis after COVID-19 vaccination (16). We deduce that the vaccine was relatively safe, and only rare cases resulted in death. Healthcare workers should pay more attention to cases of anaphylactic shock with a higher risk of death.

For most conventional vaccines such as influenza vaccine, hepatitis B vaccine, the rate of anaphylaxis after receipt of the vaccine was lower than 1 per million doses (17–20). According to the Centers for Disease Control and Prevention (CDC) (https://covid.cdc.gov/covid-data-tracker/#vaccination-trends_vacctrends-total-cum), the cumulative count of total doses administered and reported to the CDC was 340,837,941 until 11 June 2021. Based on the data of reports from VAERS, the estimated rate of anaphylaxis cases was nine per million doses, and the estimated rate of allergic reactions was 42.9 per million doses. Other reports of surveillance data show the rate of anaphylaxis for the Pfizer-BioNTech vaccine is approximate one per 200,000 doses, and the rate of anaphylaxis for the Moderna vaccine is 1 per 360,000 doses. A recent report of surveillance data shows the rate of anaphylaxis for both the mRNA vaccines is approximately at 5.5 per million doses (15). And the relative risk between COVID-19 and influenza vaccines was observed for allergic reactions by another study (21). The published results of phase 1/2 clinical trial of the inactivated SARS-CoV-2 vaccine in China showed that the most common symptom was injection-site pain (13 to 21% in different dose groups). Only one case of acute hypersensitivity manifesting as urticaria was reported (1/144) in this clinical trial (22). It is not surprising that anaphylaxis has not been reported in the clinical trials as the very low incidence and the exclusion of individuals with a past history of allergic reactions. In general, the COVID-19 vaccines are safe and the risk of hypersensitivity is perhaps a little greater compared to those of traditional vaccines (23).

A cross-sectional study about side effects of Pfizer-BioNTech COVID-19 vaccine among healthcare workers showed the prevalence of urticarial was 22.2% (24). Another study of adverse events of COVID-19 vaccines in 247 healthcare workers and medical students showed the most common systemic adverse events were fatigue, headache and muscle pain, no anaphylaxis was observed (25).

COVID-19 vaccines and other vaccines, as with other medicines, have the potency to induce hypersensitivity reactions. An active component and other attached elements of the vaccines may act as antigens. However, allergic reactions are infrequently due to the components of vaccines. Egg protein, gelatin, and other additives are the known common vaccine antigens that could induce allergic reactions after receipt of vaccines. In the majority of individuals with potential hypersensitivity to the antigens of vaccines, a lot of these antigens exist in very low levels that are commonly inadequate to cause hypersensitivity reactions. However, if there is a remarkably large amount of IgE antibody in some individuals, they can in theory respond to very low levels of these antigens and undergo serious allergic reactions, even anaphylaxis (13).

Two new lipid nanoparticles are included in the Pfizer-BioNTech vaccine; one is “pegylated” (Polyethylene glycol, molecular weight 2000 Da, PEG2000). The pegylated lipid (PEG2000) was also contained in the Moderna vaccine. Polyethylene glycol (PEG) is widely used in medicinal, cosmetic, and household products. PEGs are produced through the polymerization of ethyleneoxide, leading to PEG polymers with different molecular weight and chain length (26). Some cases of immediate-type hypersensitivity to PEGs have been reported (26, 27). PEG allergy is uncommon, and allergic reactions to PEG reported in previous published papers are owing to PEGs with high molecular weight. PEGs with low molecular weight are widely used in a lot of household products. Allergic reactions are unusually caused by these low molecular weight PEGs (26). In a previous study of patients with a past history of anaphylaxis caused by PEGs, they were performed for IgE antibodies to PEG and some had positive results (28). However, the mechanism of PEG allergy still remains indefinite. In the international consensus of recommended evaluation and management COVID-19 vaccines that recently published, for patients with a past history of a serious hypersensitivity reaction, including anaphylaxis, to a COVID-19 vaccine or its excipients, the evidence suggests against *in vitro* test or routine skin test with COVID-19 vaccines or its excipients performed by the clinician for the purpose of vaccine withholding, except for researches. The reason is that the sensitivity and specificity of the *in vitro* or skin tests in predicting serious hypersensitivity reactions such as anaphylactic reactions to COVID-19 vaccines is unclear (29). Future studies are needed to further identify the mechanism of allergy to COVID-19 vaccines and to develop efficient testing methods.

As patients may consult allergists to get additional information prior to vaccination, a suggested approach provided a framework and guidance for practicing allergists. Physicians of allergy should plan for the main population health challenges: making sure that people with high risk of allergic reactions are suitably notified and given enough support to receive the COVID-19 vaccines (30).

There are some limitations to this study. First, we can only retrospectively obtain information from the symptoms reported, and the quality and completeness of information submitted by VAERS reporters vary widely, making the assessment of causality challenging. Second, reporting biases may exist in the VAERS, and over-reporting or under-reporting may exist. Third, the accurate incidence rates of allergic reactions cannot be calculated as the total number of vaccines administered, and the number administered for each vaccine is not mentioned on the VAERS database.

In conclusion, we conducted a large sample study of allergic reactions after COVID-19 vaccination based on the VAERS database. We found a female predominance in the allergic reaction cases. A history of allergies; asthma; and anaphylaxis were risk factors for anaphylaxis post-COVID-19 vaccination. People with these risk factors should be monitored more strictly after COVID-19 vaccination.

## Data Availability Statement

The original contributions presented in the study are included in the article/Supplementary Material, further inquiries can be directed to the corresponding author/s.

## Ethics Statement

The studies involving human participants were reviewed and approved by Institutional Review Board of Peking Union Medical College Hospital. Written informed consent for participation was not provided by the participants' legal guardians/next of kin because this study was based on the Vaccine Adverse Event Reporting System (VAERS) database which publicly available and no potentially identifiable human image or data in the database.

## Author Contributions

KG and BZ conceived the work and edited the manuscript. SB did statistical analysis of the data and wrote the manuscript. LL, ZW, LC, and YX edited the manuscript. BZ conducted data mining in the VAERS database. All authors contributed to the article and approved the submitted version.

## Funding

This work was funded by the National Natural Science Foundation of China (Funding Number: 82070033).

## Conflict of Interest

The authors declare that the research was conducted in the absence of any commercial or financial relationships that could be construed as a potential conflict of interest.

## Publisher's Note

All claims expressed in this article are solely those of the authors and do not necessarily represent those of their affiliated organizations, or those of the publisher, the editors and the reviewers. Any product that may be evaluated in this article, or claim that may be made by its manufacturer, is not guaranteed or endorsed by the publisher.

## Supplementary Material

Supplemental Table 1 Features of the 11 death cases with grade 5 anaphylaxis.
