# Supplementary material for: Allergic Reactions After the Administration of COVID-19 Vaccines
Source: Front Public Health. 2022 May 17;10:878081. doi: 10.3389/fpubh.2022.878081 (PMC9152252; doi:10.3389/fpubh.2022.878081)
Supplement: Supplemental Table 1 — Features of the 11 death cases with grade 5 anaphylaxis. [file Table_1.DOCX]

Supplemental table 1. Features of the 11 death cases with grade 5 anaphylaxis.

| Age/Sex | Medical history | Symptoms | Time interval (from vaccine administration to symptoms occur) | Adrenaline received |
| --- | --- | --- | --- | --- |
| 69/F | Hypertension, Brain aneurysm | Anaphylactic reaction, dyspnea, loss of consciousness | 22 minutes | Not provided |
| NA | Not provided | Anaphylactic shock | Not provided | Not provided |
| 89/F | Not provided | Respiratory-cardio arrest; urticarial rash; vomiting; left flaccid hemiparesis | 10minutes | Not provided |
| 79/F | Not provided | Breathing problems/heart attack; anaphylactic shock | Not provided | Not provided |
| 69/F | Lewy body dementia, appendicitis, urinary incontinence and repeated urinary tract infections | Anaphylactic reaction; Acute hypoxemic respiratory failure; Cardio-respiratory arrest | 1 hour | 0.3 mg sc |
| NA | Not provided | Anaphylactic shock | Not provided | Not provided |
| 82/M | Not provided | Anaphylaxis, nausea and vomiting, shortness of breath, cough, bradycardic, lost consciousness, throat swelling, itching arm | Not provided | Not provided |
| 50/F | Not provided | Angioedema, cardiac arrest | 24 hour | Not provided |
| 81/F | Congestive heart failure, arrhythmia | Shock; Loss of consciousness; anaphylactic reaction | Not provided | Not provided |
| 60/M | Hypersensitivity | Anaphylactic shock | Not provided | Not provided |
| 27/F | Asthma, nasal polyps | GI symptoms and diarrhea, hard breathing, cardiac arrest | One the day receiving the vaccine | Yes (the dose and route was unknown) |
